# ﻿Two new species of the treehopper genus *Enchenopa* Amyot & Serville, 1843 (Hemiptera, Membracidae) from northwest Ecuador

**DOI:** 10.3897/zookeys.1216.124181

**Published:** 2024-10-21

**Authors:** María P. Rueda-Rodríguez, Jorge L. Montalvo-Salazar

**Affiliations:** 1 Universidad San Francisco de Quito USFQ, Colegio de Ciencias Biológicas y Ambientales, Instituto de Biodiversidad Tropical IBIOTROP, Laboratorio de Zoología Terrestre, Museo de Zoología, Quito 170901, Ecuador Universidad San Francisco de Quito USFQ Quito Ecuador; 2 Tandayapa Cloud Forest Station, Colegio de Ciencias Biológicas y Ambientales, Universidad San Francisco de Quito USFQ, P.O. Box 17-1200-841, Quito, Ecuador Universidad San Francisco de Quito USFQ Quito Ecuador

**Keywords:** Membracinae, Membracini, Tandayapa, taxonomy, Tropical Andes, urban green spaces

## Abstract

*Enchenopa* Amyot & Serville, 1843 is a diverse treehopper genus widespread across the New World. We describe two new *Enchenopa* species from northwest Ecuador: *Enchenopagennyae***sp. nov.** from urban forest remnants at the foothills of the Andes cordillera and *Enchenopachocoandina***sp. nov.** from secondary montane forests. *Enchenopagennyae***sp. nov.** is placed in the *E.biplaga* species group and is distinguished by the sexual dimorphism of the pronotal horn and lateral carina shape, the straight metopidium, 2–4 accessory carinae and the whitish dorsal spot and subapical band. *Enchenopachocoandina***sp. nov.** belongs to the *E.andina* species group and is diagnosed by its reddish central carina and posterior pronotal process apex, presence of an obtuse projection rather than an anterior horn, three or four irregular accessory carinae, and apical amber forewing patch. Illustrations, notes on natural history, and keys to species of the *E.biplaga* and *E.andina* species groups are also provided.

## ﻿Introduction

*Enchenopa* Amyot & Serville, 1843 is a diverse New World treehopper genus belonging to Membracinae, the second most specious subfamily of the New World Membracidae (Bartlett et al. 2018). It is differentiated from other Membracini genera by the horn-shaped anterior process or obtuse projection on the pronotum with a lateral carina running from the apex of the anterior process to, usually, the lateral margin of pronotum, and two or more pairs of accessory carinae on the metopidium (Strümpel and Strümpel 2014). *Enchenopa* is most similar to *Enchophyllum* Amyot & Serville, 1843 due to the pronotal horn and lateral carinae, but it differs mainly in the presence of the accessory carinae and the lateral carina usually surpassing the humeral angles. *Enchenopa* can be distinguished from *Membracis* Fabricius, 1775, *Folicarina* Sakakibara, 1992, and *Phyllotropis* Stål, 1869 by the non-foliaceus pronotum, presence of a pronotal horn, and the carination which is typically absent in *Membracis* and reduced in *Folicarina* and *Phyllotropis*; although *Folicarina* also has accessory carinae (Flórez-V et al. 2015; McKamey 2022). Richter (1947, 1954) noted that some *Membracis* species occasionally have a short lateral carina and, *Enchophyllum* and *Enchenopa* can vary intraspecifically in the length of the lateral carina. Lastly, *Enchenopa* can only be differentiated accurately from *Leioscyta* Fowler, 1894 by the accessory carinae, which are absent in the latter. Some *Leioscyta* are also smaller species, lack lateral carinae, and the pronotum is rounded (Dietrich and McKamey 1995). *Enchenopa* is not supported by any synapomorphies, and phylogenetic studies have suggested that it, as well as its related genera, may not be monophyletic (Dietrich and McKamey 1995; Lin et al. 2004).

Strümpel and Strümpel (2014) revised *Enchenopa* and recognized 51 valid species, including 21 new to science, and classified them into ten species groups. They also treated *Campylenchia* Stål, 1869 as a junior synonym. Since then, only one more species of the *E.andina* species group has been described from Brazil (Lencioni-Neto and Sakakibara 2015). McKamey (2022) reinstated four more species excluded from the genus by Strümpel and Strümpel (2014) however, the types are not in a good state of preservation and he could not evaluate possible synonymy.

The biology of most species of *Enchenopa* is poorly known. Some species are solitary but occasionally congregations of adults with nymphs can be found. There is no parental care; instead, the females deposit their eggs in clusters on their host plant covered with a white wax-like substance that protects them from parasitoids (Godoy et al. 2006; Lin 2006). They have been reported to have mutualistic relationships with ants and to feed on host plants from at least 32 families of which Fabaceae and Asteraceae are the most predominant (Flórez-V et al. 2015). *Enchenopabinotata* (Say, 1824) is undoubtedly the most studied species of the genus, and its mating signals and host plant specialization suggest that this taxon comprises a complex of as many as 15 species (McNett and Cocroft 2008; Hamilton and Cocroft 2009; Deitz and Wallace 2012).

Species of *Enchenopa* are distributed from Canada to Argentina, but most species inhabit the Neotropical region. Strümpel and Strümpel (2014) recorded 12 species in Ecuador. Nevertheless, they could not review enough material, especially, from the Andean region. Previously Goding (1928) registered the species *E.lanceolata* (Fabricius, 1787), *E.quadricolor* (Walker, 1858), *E.minans* (Fairmaire, 1846) and *E.tatei* (Goding, 1928) in Ecuador not recorded by Strümpel and Strümpel (2014).

In this study, we describe two new species of *Enchenopa* from northwestern Ecuador, one of which was found in the forest remnants of a populous city at the foothills of the Andean Cordillera, and the second species from secondary montane forests. Additionally, we provide keys to species of the *Enchenopabiplaga* and *E.andina* species groups to which the newly described species belong.

## ﻿Materials and methods

Fieldwork was conducted between 2023 and 2024 in two locations in northwest Ecuador, urban forest remnants of Santo Domingo (Santo Domingo de los Tsáchilas) composed of patches of secondary semi-deciduous forest and located next to water bodies between 300 and 600 m of elevation, and Tandayapa Cloud Forest Station (Pichincha), a scientific station of Universidad San Francisco de Quito founded in 2022 and located in the Tandayapa Valley at 2280 m of elevation. The station covers 53 hectares of secondary and mature Montane Forest. Specimens were collected opportunistically during the day and euthanized in 75% ethanol. Observations of natural history were recorded in situ. A light trap with a mercury lamp was set up in Tandayapa Cloud Forest Station from 6 pm to 6 m in February 2024, and all membracid specimens were collected. We also examined specimens deposited at the Museo de Zoología Universidad San Francisco de Quito, Ecuador (ZSFQ), where all our specimens are deposited.

The specimens were photographed and measured using an Olympus DP73 digital camera attached to the Olympus SZX16 stereomicroscope with the light diffused as adapted from Kerr et al. (2008). The measurements were taken following Strümpel and Strümpel (2014). To examine the genitalia, we removed the entire abdomens and cleared them using 10% KOH for 48 hours at room temperature and then washed with distilled water. Genitalia were photographed using an OMAX A35180U3 digital camera attached to Olympus CX22 optic microscope and afterwards preserved in a 0.2-ml microvial with glycerol pinned with their respective specimen. Images were compiled in one multifocal composition using Zerene Stacker - USDA SI-SEL Lab Bk imaging system. The illustrations were made digitally with Sketchbook, a free access illustration software. Final images were edited in Adobe Photoshop CC 2023.

Terminology of general morphology follows Deitz (1975), Dietrich et al. (2001), and Strümpel and Strümpel (2006, 2014).

## ﻿Results

### ﻿Key to species of *Enchenopabiplaga* species group (modified from Strümpel and Strümpel 2014)

**Table:** 

1	Pronotum with one white or yellow dorsal spot	**2**
–	Pronotum with one white or yellow dorsal spot and one white or yellow subapical band	**3**
2	Pronotal horn short, curved forward, dorsal spot always yellow	***E.ignidorsum* Walker**
–	Pronotal horn long and straight, white or yellow dorsal spot	***E.vittifera* Stål**
3	Pronotum with horn length shorter than distance between tips of humeral angles or horn absent	**4**
–	Pronotum with horn longer than distance between tips of humeral angles	**7**
4	Metopidium with two accessory carinae, the posterior one half to almost as long as lateral carina	**5**
–	Metopidium with two to four accessory carinae, all < 1/2 length of lateral carina	**6**
5	Pronotal horn curved forwards; lateral carina just surpassing the corner of humeral angles; posterior accessory carinae almost length of lateral carina. Males / females with dorsal spot 2 × / 4 × as long as subapical band	***E.longimaculata* Strümpel & Strümpel**
–	Pronotal horn straight; lateral carina almost touching lateral pronotal margin; posterior accessory carinae 1/2 length of lateral carina; dorsal spot and lateral band not sexually dimorphic	***E.singularis* Strümpel & Strümpel**
6	Metopidium convex and two to three accessory carinae. Male and female not dimorphic. Overall color brown to black	***E.biplaga* Walker**
–	Metopidium straight with two to four accessory carinae. Male with narrow horn curved forwards and female with only obtuse projection; male shorter than female. Overall color black	***E.gennyae* sp. nov.**
7	Pronotum with horn curved, directed forward	**8**
–	Pronotum with horn straight	**9**
8	Length from pronotum base to horn apex equal to distance from pronotum base to posterior apex of pronotum	***E.dubia* (Fowler)**
–	Length from pronotum base to horn apex shorter than distance from pronotum base to posterior apex of pronotum	***E.lanceolata* Fabricius**
9	Pronotum with horn distinctly longer than body width, lateral carina just surpassing horn base	***E.reticornuta* Strümpel & Strümpel**
–	Pronotum with horn slightly longer than body width, lateral carina almost touching lateral pronotum margin	***E.richteri* Strümpel & Strümpel**

#### ﻿ Enchenopa gennyae sp. nov.

Taxon classificationAnimaliaHemipteraMembracidae

3BEBA66A-A6EC-5F29-9C22-0F61A60AB3A0

https://zoobank.org/F7605C6C-FB5D-4337-A24E-84A915713DC4

Figs 1 , 2 , 3 , 4

##### Material examined.

***Holotype***: Ecuador • 1 ♀; Santo Domingo de los Tsáchilas, Santo Domingo, Río Baba -0.30295, -79.15211, 480 m; 12 May 2023; Montalvo, J. & Rueda, M. P. leg.; Ex. Manual ZSFQ-i12112. ***Paratypes***: Ecuador • 2 ♂; same labels as for holotype; ZSFQ-i12110, ZSFQ-i12111 • 2 ♀, 1 ♂ Santo Domingo de los Tsáchilas, Santo Domingo, Quebrada del Río Pove -0.25237, -79.156668, 570 m; 14 Aug. 2023; Rueda, M. P. & Montalvo, J. leg.; Ex. Manual; ZSFQ-i17766:17768 • 3 ♀, 1 ♂ same locality as paratypes; 20 Apr. 2024; Rueda, M. P. leg.; Ex. Manual; ZSFQ-i18855:18858.

##### Notes on the type series.

All specimens are minuten-mounted. Dissected abdomens of the holotype and two female and two male paratypes were placed in vials with glycerol pinned beneath the specimens. Some paratypes are in poor condition, with legs or wings lost.

##### Additional material.

Ecuador • 3 5^th^ instars; same data as paratypes; ZSFQ-i17925, 17926.

##### Diagnosis.

Overall color black with whitish dorsal spot and subapical band; metopidium straight, two to four sub-equal accessory carinae. Sexually dimorphic: female with obtuse projection instead of horn, short lateral carina not surpassing humeral angles, dorsal spot 2× longer than subapical band, and longer than male in size; male with narrow horn slightly curved forwards, lateral carinae almost touching lateral margin of pronotum and dorsal spot subequal in length to subapical band.

##### Description.

**Female holotype** (ZSFQ-i12112). ***Measurements*** (mm): Length from head to wings at rest: 5.7; Total length: 6.3; Head to apex of posterior process: 4.6; Pronotal length: 4.4; Head to horn apex: 2.4; Forewing length: 4.9; Body width: 1.9; Vertex width on ocellar line: 1; Head length: 1.2; Frontoclypeus length: 0.8; Frontoclypeus width: 0.7; Prothoracic tibia length: 0.9; Metathoracic tibia length: 1.4; Metathoracic tibia width: 0.3; Prothoracic tibia width: 0.3.

***Color*.** Overall black with whitish dorsal spot and subapical band on dorsum. Dorsal spot 2× longer than subapical band. Eyes black with dark brown margins, ocelli golden. Forewings opaque dull black, hind wings hyaline, veins black. Tarsi golden.

***Surface*.** Head, pronotum, ventral sclerites of thorax, legs, and abdomen with dense golden pubescence; subcostal cell and veins of forewings with short, dispersed, almost indistinguishable golden pubescence. Pronotum (except metopidium) strongly punctured. Head, metopidium, forewings, and legs rough.

**Figure 1.**
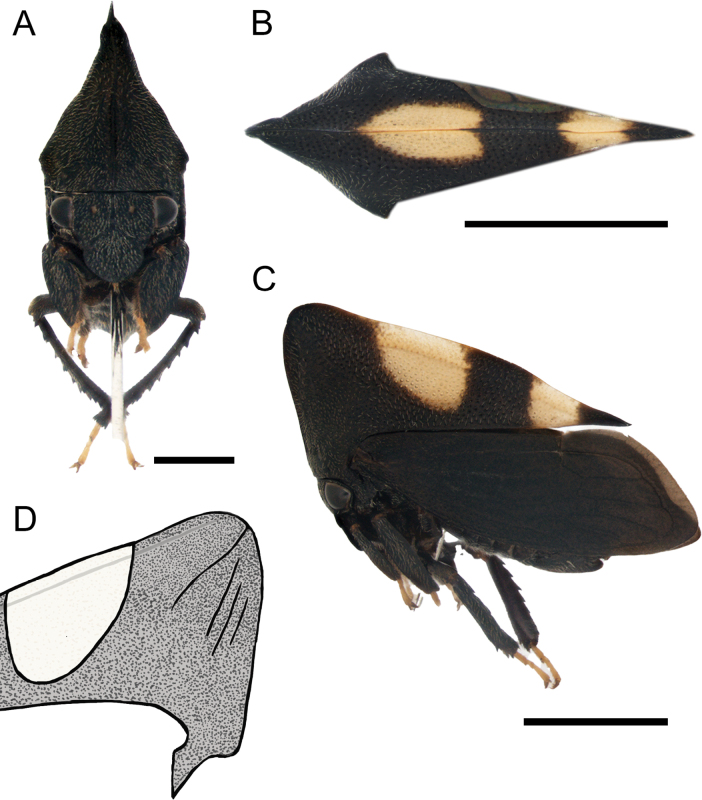
*Enchenopagennyae* sp. nov. holotype female **A–C** habitus in frontal, dorsal view, and lateral views respectively **D** illustration of pronotum showing the carination. Scale bars: 1 mm (**A**); 2 mm (**B, C**).

***Head*.** Triangular, longer than wide (avoiding eyes); ocelli closer to eyes than each other; supra-antennal ledges arranged above clypeus; clypeus broad, longer than wide, anterior margin rounded; rostrum reaching hind coxae (Fig. 1A).

***Thorax*.** Pronotum somewhat compressed, in lateral view, triangular, dorsal contour arched; metopidium straight, inclined anteriorly; horn reduced to an obtuse projection, obliquely directed dorso-anteriorly, wider than long, apex broadly rounded; median carina laminated and somewhat foliaceous, especially on dorsum; lateral carina short, not extending beyond humeral angles (Fig. 1C); three parallel accessory carinae, almost as long as lateral carina extending ventroposteriorly from projection apex; posterior apex of pronotum acuminate almost reaching the apex of first apical cell (Fig. 1D); humeral angles slightly produced (Fig. 1B). Forewings with five apical cells, one discoidal cell, and one vein in the clavus, one r-m crossvein, two m-cu crossveins, and without s crossvein; apical limbus broad. Hind wings with four apical cells and one r-m crossvein. Anterior and middle tibiae foliaceous; posterior femur with apical, ventral and middle cucullate setae, metathoracic tibia compressed with spine-like cucullate setae on rows I and II, row III absent.

**Figure 2.**
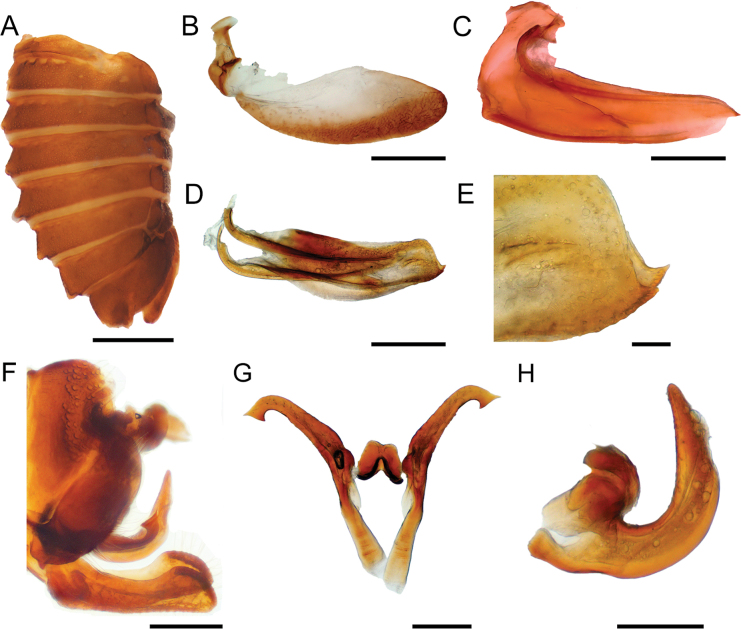
Abdomen and terminalia of *Enchenopagennyae* sp. nov. **A** undissected female abdomen in lateral view **B** gonoplac in lateral view **C** first valvula in lateral view **D** second valvula in lateral view **E** close-up apex of second valvula **F** undissected male pygofer in lateral view **G** styles in dorsal view **H** aedeagus in lateral view. Scale bars: 1 mm (**A**); 0.1 mm (**B–D**); 0.01 mm (**E**); 0.2 mm (**F**); 0.05 mm (**G, H**).

***Abdomen*.** Sternum III with a transverse keel extending along the sternite and slightly projected ventrally. Dorsum of tergites VII and VIII with medial tuberosities, tergites IV–VI with reduced medial tuberosities (Fig. 2A). Genitalia. Gonoplac ventrally with few setae and more sclerotized, apex rounded (Fig. 2B). First valvula blade shaped, apex ventrally rounded, dorsally acuminate extending beyond ventral margin (Fig. 2C). Second valvula broad throughout, dorsally rounded, ventrally weakly serrated with a ventral apical tooth directed upwards (Fig. 2D, E).

**Male paratype** (ZSFQi-17766). Similar to female except dorsal spot as long as subapical band, pronotal horn narrow and curved forwards, lateral carina almost touching lateral margin of pronotum, metopidium with three accessory carinae at each side. Genitalia. Subgenital plate, in lateral view, 3× longer than wide, lobes diverging in first 1/4, dorsal margin concave, distally expanded (Fig. 2F). Styles 5× as long as wide, anterior projection subequal to posterior projection; shank with notch at middle of ventral margin, distally recurved and apically truncate, slightly expanded with posterior end longer and narrower than anterior end (Fig. 2G). Aedeagus U-shaped with anterior arm smaller than posterior arm and rounded; posterior arm lanceolate and abruptly narrowed at 1/3 length, anterior surface smooth, without serrations (Fig. 2H).

##### Variation.

***Measurements*.** Male / Female (mm): Length from head to wings: 4.4–5.1 / 5.3–5.8; Total length: 4.9–5.6 / 6.2–6.3; Head to apex of posterior process: 3.8–4.5 / 4.58–5; Pronotal length: 3.8–5.5 / 4.4–5.3; Head to horn apex: 2.2–2.7 / 2.4–2.9; Forewing length: 3.7–4.2 / 4.5–5.1; Body width: 1.6–1.7 / 1.9–2; Vertex width on ocellar line: 0.9–1 / 1.0–1.1; Head length: 1.1–1.2 / 1–1.2; Frontoclypeus length: 0.7–0.8 / 0.7–0.8; Frontolypeus width: 0.7 / 0.7–0.8; Metathoracic tibia length: 0.8–1 / 0.9–1; Prothoracic tibia length: 1.2–1.4 / 1.4–1.8; Metathoracic tibia width: 0.2–0.3 / 0.3–0.5; Prothoracic tibia width: 0.3–0.4 / 0.3.

**Figure 3.**
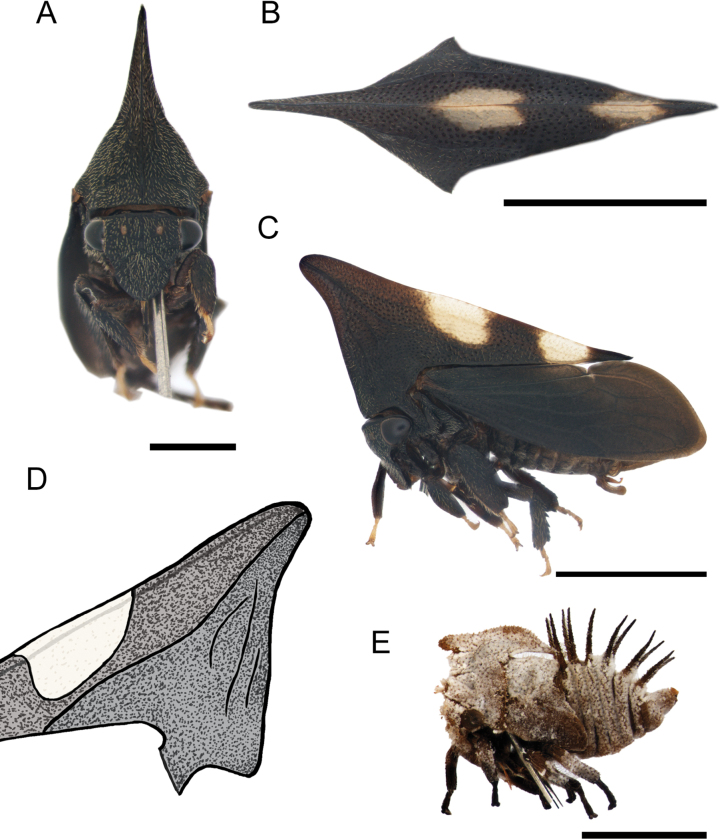
*Enchenopagennyae* sp. nov. paratype male and nymph **A–C** male habitus in frontal, dorsal view, and lateral views respectively **D** illustration of male pronotum showing the carination **E** nymph habitus in lateral view. Scale bars: 1 mm (**A**); 2 mm (**B, C, E**).

Females longer than males, with obtuse projection instead of horn, dorsal spot 2× longer than subapical band, lateral carina not surpassing humeral angles and, in some individuals, weakly produced; two to four secondary carinae almost as long as lateral carina. Male with horn narrow and curved forwards, dorsal spot < 2× subapical band length (Fig. 3A–C), lateral carina almost attaining lateral margin of pronotum and metopidum with three accessory carinae on each side (Fig. 3D). Independent of gender, dorsal contour of pronotum is more or less arched.

##### Fifth-instar nymph description.

Overall color mostly white with black tarsi and scoli (Fig. 3E). One pair of abdominal scoli on each segment from III to VII; scoli length 5–6× basal width. Pronotum anteriorly with nascent horn not extended beyond head and posteriorly extended to abdominal segment III; anterior apex rounded and directed forward; posterior apex acute, dorsal margin convex in middle. Needle-like setae on chalazae distributed over whole body.

##### Distribution and natural history.

**Figure 4.**
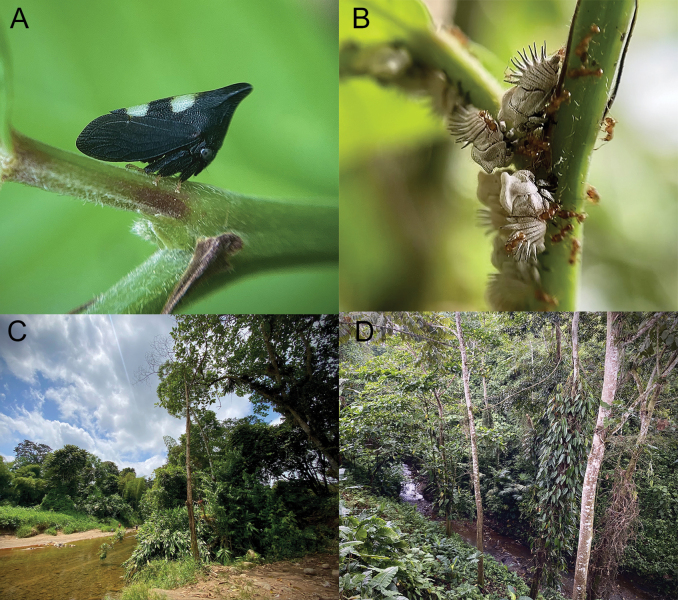
*Enchenopagennyae* sp. nov. in its natural environment and habitat **A** male paratype perched on a stem of its host plant **B** nymphs attended by *Wasmanniaauropunctata* (Roger) **C** shore of Baba River **D** ravine of Pove River.

Specimens of *Enchenopagennyae* sp. nov. were found in two secondary forest remnants of the Western Foothills Forest from the urban area of ​​Santo Domingo (Fig. 9): on the banks of the Baba River (Fig. 4C) and Pove River’s ravine (Fig. 4D). Adult and nymph congregations were found on several occasions cohabiting together and perched on the underside of leaves and stems of an unidentified species of the genus *Piper* L. between 100 and 150 cm above the ground (Fig. 4A). Females were always more abundant than males in these congregations. Nymphs were attended by fire ants of the species *Wasmanniaauropunctata* (Roger) (Fig. 4B).

##### Etymology.

The species is dedicated to the mother of the first author, Genny Elizabeth Rodríguez Cueva, who helped to find the specimens of this species and has been a great support and inspiration throughout her life.

##### Remarks.

Females of *Enchenopagennyae* sp. nov. have a short lateral carina that does not extend beyond the humeral angles, while males have a long lateral carina that almost reaches the lateral margin of pronotum. In the tribe Membracini, the length of the lateral carina has not been previously reported as sexually dimorphic in any species. However, in some species of *Membracis*, it has been noted that the lateral carina may or may not be present among individuals (Richter 1947). Likewise, Richter (1954) argued the lateral carina of some species of *Enchophyllum* and *Enchenopa* can vary in length within the same population. This species is the first known in the *Enchenopabiplaga* species group to exhibit sexual dimorphism in pronotum shape and lateral carina length. Like *Enchenopagennyae* sp. nov., *E.longimaculata* Strümpel & Strümpel, 2014 has remarkable sexual dimorphism of the dorsal spot. However, in *Enchenopalongimaculata* the females the spot is 4× the length of the subapical band and in males 2× while in *E.gennyae* sp. nov. the spot is 2× as long in females but subequal in males.

Sexual dimorphism in the shape of the pronotal horn is characteristic of the *Enchenopaminuta* species group. However, *Enchenopagennyae* sp. nov. does not belong to that group, given its coloration, the shape of the second valvula and, more importantly, the female’s lack of a pronotal horn; in the *E.minuta* species group, males lack the horn and females generally have a developed horn.

*Enchenopagennyae* sp. nov. belongs to the *E.biplaga* species group due to the presence of bands on dorsum of the pronotum, the second valvula with a ventral apical tooth, and forewings with one discoidal cell. *Enchenopagennyae* sp. nov. differs from *E.ignidorsum* (Walker, 1858) and *E.vittifera* (Stål, 1869) by the two white lateral bands instead of just one yellow or white one, respectively. *Enchenopagennyae* sp. nov. differs from *E.dubia* (Fowler, 1894), *E.lanceolata*, *E.reticornuta* Strümpel & Strümpel, 2014, and *E.richteri* Strümpel & Strümpel, 2014 by a horn shorter than the distance between the tips of humeral angles rather than longer. *Enchenopasingularis* Strümpel & Strümpel, 2014 and *E.longimaculata* have two accessory carinae with the posterior one ~ 1/2 or almost the total length, respectively, of the lateral carina, while *E.gennyae* sp. nov. has from two to four somewhat subequal accessory carinae. *E.gennyae* sp. nov. is distinguished from *E.melaleuca* Walker, 1858 by the shorter horn that is not curved forward. The new species closely resembles *Enchenopabiplaga* Walker, 1858 due to the shape of the pronotal horn, pronotum coloration, and the disposition of the accessory carinae. However, *Enchenopagennyae* sp. nov. has a straight metopidium, less produced horn, and sexual dimorphism. In contrast, in *E.biplaga* the metopidium is convex, the horn is large and strongly produced, and without sexual dimorphism. The females of *E.gennyae* sp. nov. have a short and straight horn while *E.biplaga* females have the horn longer than wide and curved forward. In males of *E.gennyae* sp. nov. the posterior arm of the aedeagus, in lateral view, is abruptly narrowed at one-third of its length, and the apical hook of the styles has the posterior tooth longer and narrower than the anterior tooth. In contrast, in the male of *E.biplaga* the width of the posterior arm of the aedeagus, in lateral view, slightly decreases at half of its length, and the apical hook of the styles has the posterior tooth similar in size to the anterior tooth.

McKamey (2022) reinstated *Enchenopamelaleuca* from the genus *Enchophyllum* and suggested it shares morphological similarities with some species of the *E.biplaga* species group. However, the holotype of this species has yet to be reviewed to confirm these affinities. Therefore, excluded *E.melaleuca* from the key to *E.biplaga* group species but compared it with *E.gennyae* sp. nov. in the above discussion.

In the *E.biplaga* species group, nymphs of *E.reticornuta* and *E.vittifera* are known (Strümpel and Strümpel 2014). They share with the nymph of *E.gennyae* sp. nov. the body covered with white wax-like material, black tarsi, and the presence of scoli on abdominal tergites III–VIII. However, we found the nymphs of *E.gennyae* sp. nov. differ from them mainly in the shape of the pronotum and scoli. The nymphs of *Enchenopavittifera* have a longer horn directed forward with the posterior apex of the pronotum reaching the abdominal tergite III and have shorter scoli. While the nymphs of *Enchenopareticornuta* have a straight horn with the posterior apex of the pronotum not touching the abdomen and longer scoli widened at the base.

### ﻿Key of species of *Enchenopaandina* group (modified from Strümpel and Strümpel 2014; Lencioni-Neto and Sakakibara 2015)

**Table:** 

1	Pronotal horn horizontally directed forwards	***E.loranthacina* Sakakibara & Marques**
–	Pronotal horn obliquely directed upwards and forwards or horn absent and replaced by an obtuse projection	**2**
2	Head as long as wide or wider than long	**3**
–	Head longer than wide	**4**
3	Head as long as wide, body with long pubescence, forewings with a medial pale patch	***E.pilosa* Strümpel & Strümpel**
–	Head wider than long, body with short pubescence, forewings without a medial pale patch	***E.eurycephala* Strümpel & Strümpel**
4	Median carina or just posterior apex of pronotum reddish, apical 1/3 of forewings amber	**5**
–	Median carina and posterior apex of pronotum concolorous, forewing with hyaline patch at apical margin	**6**
5	Pronotal horn well produced; two to three accessory carinae well developed	***E.andina* Schmidt**
–	Pronotal horn absent, replaced by obtuse projection; three to four weak and irregular accessory carinae present, some touching lateral carina	***E.chocoandina* sp. nov.**
6	Pronotal accessory carinae well developed; forewing apical patch occupying all of distal margin and extended basad to middle of apical cells 3 and 4	***E.monoceros* (Germar)**
–	Pronotum with accessory carinae weak; forewing apical patch small, occupying only part of limbus	***E.luizae* Lencioni-Neto & Sakakibara**

#### ﻿ Enchenopa chocoandina sp. nov.

Taxon classificationAnimaliaHemipteraMembracidae

91C5558F-607B-5F62-9E5C-D8719E55B6AB

https://zoobank.org/BA0A3604-4903-4D28-B991-E3E3BB0365B2

Figs 5 , 6 , 7 , 8

##### Material examined.

***Holotype***: Ecuador • 1 ♂; Pichincha, Tandayapa Cloud Forest Station -0.009645, -78.688058, 2280 m of elevation; 3 Fbr. 2024; Rueda, M. P. leg.; Ex. Manual; ZSFQ-i18060. ***Paratypes***: Ecuador • 1 ♂; same data as for holotype; ZSFQ-i18061 • 3 ♀, 1 ♂; Pichincha, Tandayapa Cloud Forest Station -0.009645, -78.688058, 2280 m of elevation; 9 Fbr. 2024; López-García, M. M., Montalvo, J. & Rueda, M. P. leg.; Ex. Mercury light; ZSFQ-i10862: 10865 • 1 ♀; Pichincha, Mindo, 0.04166, -78.77472, 1300 m of elevation; 11 Jun. 2022; Torres, D. leg.; Ex. Manual; ZSFQ-i8423 • 1 ♀, Imbabura, Seis de Julio de Cuellaje, 0.4509352, -78.525948, 2000 m of elevation; 13 Nov. 2021; Rubio, A. leg.; Ex. Manual; ZSFQ-i8196.

##### Note on the type series.

Holotype and most paratypes are minuten-mounted. The paratype female ZSFQ-i8243 was originally pinned, but later the specimen was transferred to double mounting on a minuten pin. Dissected abdomens of holotype, one male paratype, and three female specimens placed in vials with glycerol pinned with specimens.

##### Diagnosis.

Overall coloration black with scarlet median carina and posterior apex in females and scarlet posterior apex in males, apical 1/3 of forewing amber; pronotal horn absent, replaced by obtuse projection directed obliquely forwards, lateral carina almost touching lateral margin of pronotum; three or four weak accessory carinae, some touching lateral carinae or bifurcate.

##### Description.

**Male holotype** (ZSFQ-i10860): ***Measurements*** (mm): Length from head to wings: 5.3; Total length: 5.4; Head to apex of posterior process: 4.5; Pronotal length: 4.6; Head to horn apex: 1.5; Forewing length: 4.4; Body width: 2.2; Vertex width on ocellar line: 1.2; Head length: 1.2; Frontoclypeus length: 0.7; Frontoclypeus width: 0.1; Metathoracic tibia length: 0.9; Prothoracic tibia length: 1.8; Metathoracic tibia width: 0.3; Prothoracic tibia width: 0.2.

***Color*.** Overall color black. Eyes brownish, ocelli golden. Posterior apex of pronotum scarlet red. Forewings almost entirely opaque black with an apical translucent amber patch restricted on the third to fifth apical cells and limbus around this area. Tarsi pale brownish.

**Figure 5.**
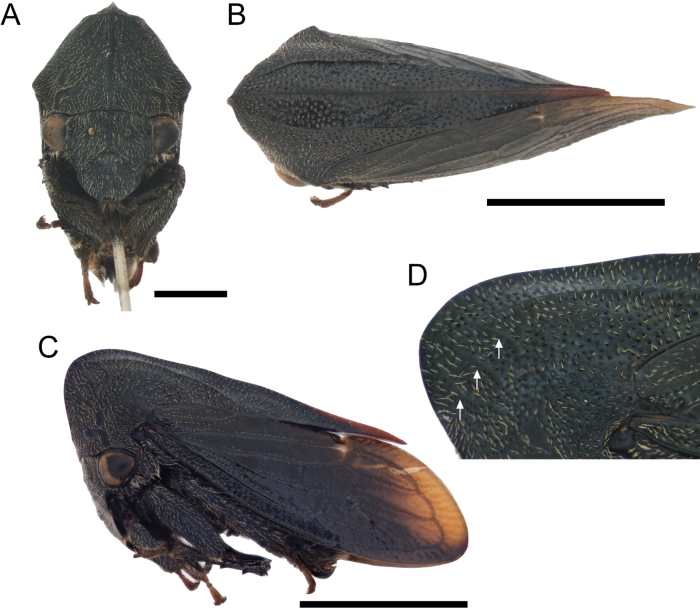
*Enchenopachocoandina* sp. nov. holotype male **A–C** habitus in frontal, dorsal view, and lateral views respectively **D** approach to base of horn in lateral view, the white arrows indicate the accessory carinae. Scale bars: 1 mm (**A**); 2 mm (**B, C**).

***Surface*.** Head, pronotum, ventral sclerites of thorax, legs, and abdomen with golden pubescence (Fig. 5B); sclerotized area of forewings with shorter pubescence. Pronotum (except by metopidium) and sclerotized area of forewings strongly punctured. Head, metopidium, legs, and abdomen rough.

***Head*.** Triangular blunt, longer than wide (excluding eyes); distance between ocelli subequal to ocelli-eye distance; supra-antennal ledges arranged above clypeus; clypeus broad, longer than wide, anterior margin rounded; rostrum reaching posterior coxae (Fig. 5A).

***Thorax*.** Pronotum, in lateral view, triangular; metopidium straight directed forwards; pronotal horn absent instead an obtuse projection with rounded apex; humeral angles slightly produced (Fig. 5B); median carina sharp; lateral carina parallel to median carina, running from apex of anterior projection to middle of the lateral margin of pronotum, almost touching the margin (Fig. 5C); three accessory carinae short (1/10 length of lateral carina), weak, irregular, sub-perpendicular to primary lateral carina, the last two diverging from the lateral carina (Fig. 5D), left side with the anterior two accessory carinae convergent; posterior apex of pronotum acuminate, just surpassing first apical cell. Forewing with one vein on clavus, two discoidal cells, two m-cu cross veins, one s cross veins which enclose second discoidal cell, and five apical cells; apical limbus broad. Anterior and middle tibiae foliaceous; posterior femur with apical ventral and middle cucullate setae, posterior tibia with spine-like cucullate setae on rows I and II, row III absent.

**Figure 6.**
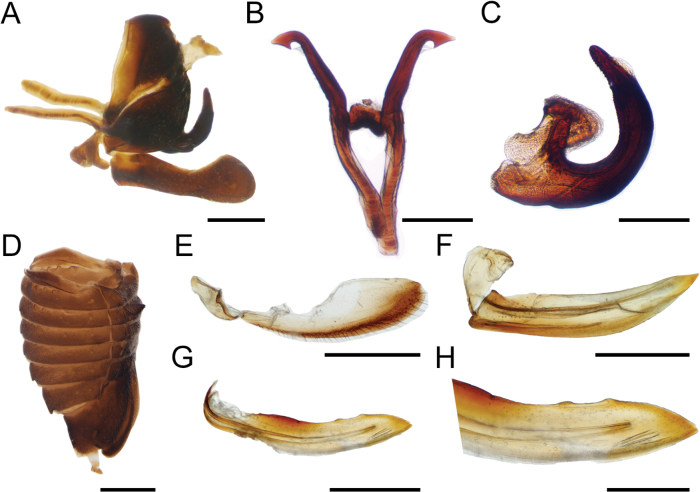
Abdomen and terminalia of *Enchenopachocoandina* sp. nov. **A** undissected male pygofer in lateral view **B** styles in dorsal view **C** aedeagus in lateral view **D** undissected female abdomen in lateral view **E** gonoplac in lateral view **F** first valvula in lateral view **G** second valvula in lateral view **H** close-up apex of second valvula. Scale bars: 0.1 (**A, H**); 0.05 (**B, C**); 1 mm (**D**); 0.2 mm (**E–G**).

***Abdomen*.** Sternum III with a transversal keel extended along the sternite, strongly projected downwards and medially invaginated. Tergites III to VI with a pair of medial tuberosities, tuberosities of tergite VI strongly developed (Fig. 6D). Subgenital plate, in lateral view, 3× longer than wide, lobes diverging since the base, dorsal margin concave (Fig. 6A). Aedeagus with posterior arm lanceolate, 2× longer than anterior arm and strongly inclined forwards; anterior face of posterior arm armed at apical1/3 with small dorsal apical denticles, gonopore subapically (Fig. 6C). Styles distally recurved, apically truncate with anterior part longer than posterior part, spine tuft on dorsal margin just anterior to apex (Fig. 6B).

**Female paratype** (ZSFQ-i8423): Similar to male except for the pronotal projection more angulated and produced, central carina reddish behind humeral angles and forewings with amber patch extended from the second to fifth apical cells and limbus around this area. Genitalia. Gonoplac ventrally setose and more sclerotized than dorsally (Fig. 6E). First valvula blade shaped, basal 2/3 broad, apex acuminate (Fig. 6F). Second valvulae blade shaped with two dorsal tubercles in apical 1/2 (Fig. 6G, H).

**Nymph** unknown.

##### Variation.

Measurements. Female / male (mm): Length from head to wings: 5.7–5.9 / 5.3–5.5; Total length: 5.9–6.4 / 5.4–5.7; Head to apex of posterior process: 5–5.6 / 4.3–4.5; Pronotal length: 4.9–5.5 / 4.5–4.6; Head to horn apex: 1.6–1.9 / 1.5–1.8; Forewing length: 5–5.4 / 4.4–4.8; Body width: 2.3–2.7 / 2.1–2.2; Vertex width on ocellar line: 1.2–1.4 / 1.1–1.2; Head length: 1.3–1.5 / 1.0–1.2; Frontoclypeus length: 0.7–0.9 / 0.7–0.8; Frontoclypeus width: 0.8–1.0 / 0.7–0.9; Metathoracic tibia length: 1–1.2 / 0.9–1.1; Prothoracic tibial length: 1.5–1.9 / 1.8–1.9; Metathoracic tibia width: 0.3–0.4 / 0.3–0.3; Prothoracic tibia width: 0.2–0.4 / 0.2–0.3.

**Figure 7.**
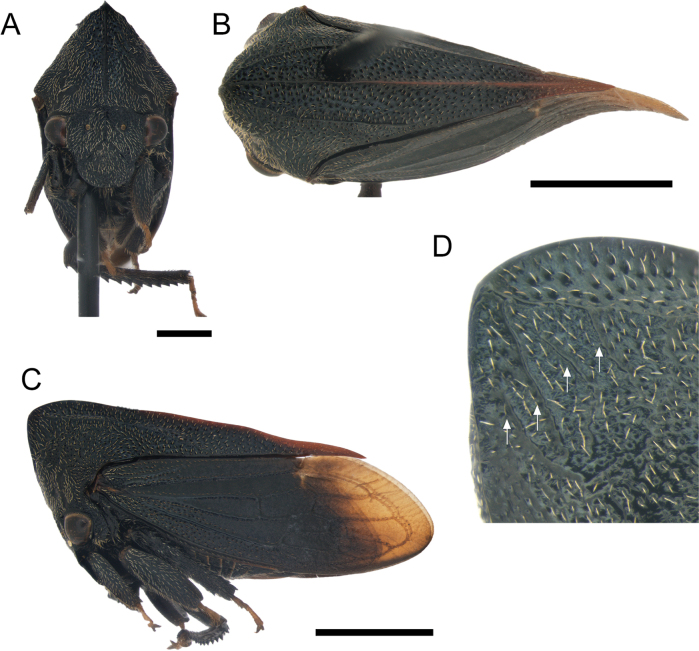
*Enchenopachocoandina* sp. nov. paratype female **A–C** habitus in frontal, dorsal view, and lateral views respectively **D** approach to base of horn in lateral view, the white arrows indicate the accessory carinae. Scale bars: 1 mm (**A**); 2 mm (**B, C**).

Females are longer and have more produced pronotal projections than males (Fig. 7A, C), three or four accessory carinae (Fig. 7D), lateral carina behind humeral angles reddish rather than just the posterior apex of pronotum, and the amber membrane is most extended (Fig. 7B). Independent of gender, some accessory carinae are bifurcate or converged.

##### Distribution and natural history.

**Figure 8.**
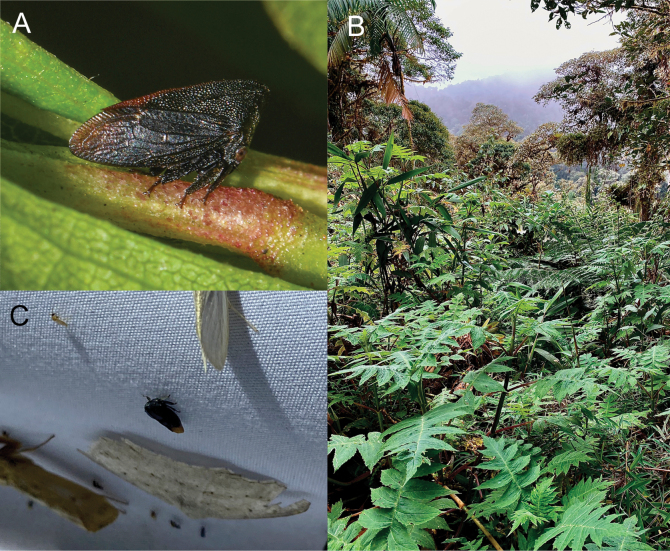
*Enchenopachocoandina* sp. nov. in its natural environment and habitat **A** paratype female from Mindo by David Torres **B** border of a secondary forest at Tandayapa Cloud Forest Station **C** female paratype attracted by a light trap.

**Figure 9.**
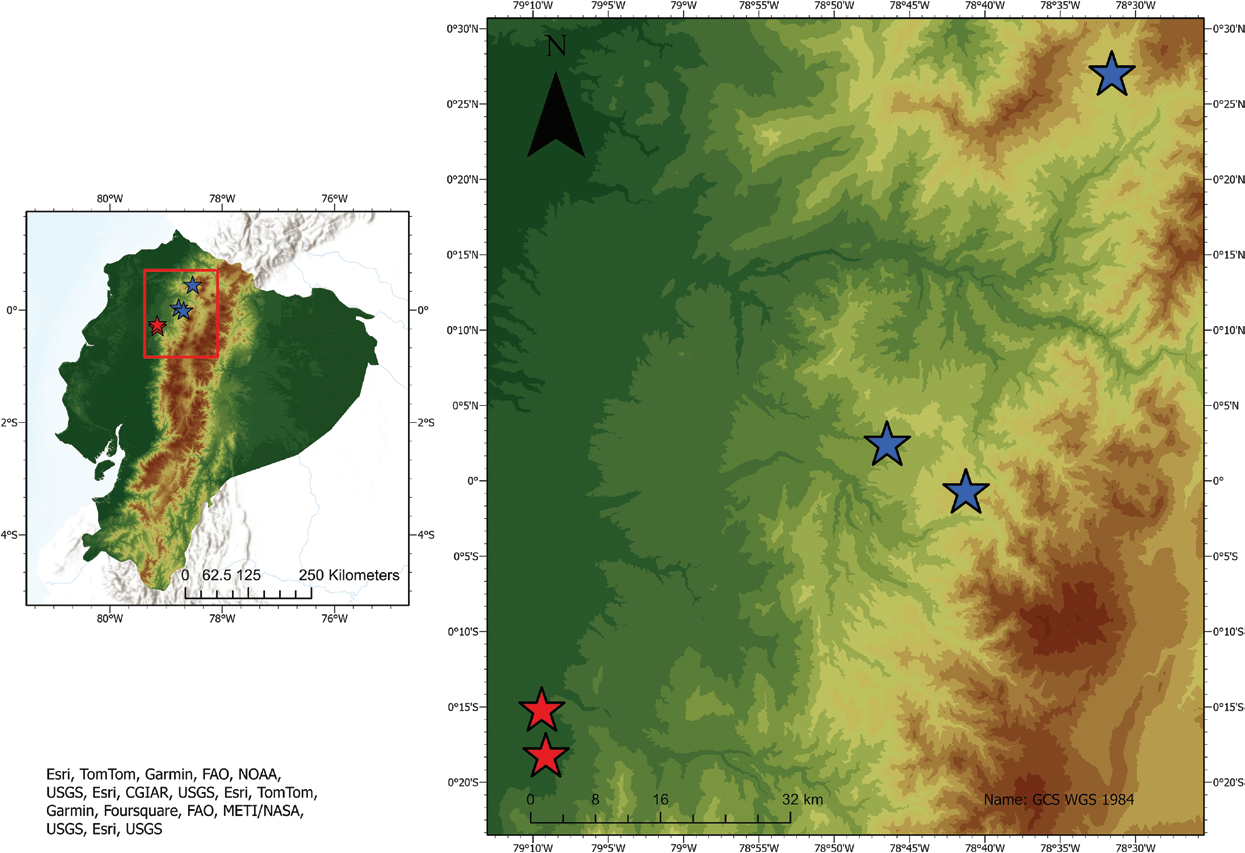
Distribution of the new species in Ecuador. Red stars = records of *Enchenopagennyae* sp. nov.; blue stars = records of *Enchenopachocoandina* sp. nov.

This species is distributed in the Montane forests of northwest Ecuadorian Andes (Fig. 9), between 1300 to 2300 m elevation. It inhabits the borders of secondary forests (Fig. 8B) and is a solitary species. The species has been recorded perched on the leaves or stems of different species of Asteraceae and Araceae, but more oftenly on *Munnoziapinnatipartita* (Hieron.) H.Rob. & Brettell (Asteraceae) (Fig. 8A), an endemic Ecuadorian species (Barriga et al. 2011). The species has been observed active during the day and attracted to mercury light traps at night between 8 pm and 2 am (Fig. 8C).

##### Etymology.

The species, a noun in apposition, is named after the Andean Choco Biosphere Reserve declared by UNESCO as the seventh biosphere reserve of Ecuador, where this species lives. It honors the people who defend this territory from the metal mining that threatens the ecosystems and biodiversity of this important area.

##### Remarks.

*Enchenopachocoandina* sp. nov. belongs to *E.andina* species group based on the pronotum with a horn or projection shorter than the distance between the humeral angles, the forewing with transparent patches, and the blade-shaped second valvulae. Moreover, some species of this group, such as *E.pilosa* and *E.eurycephala*, have dense pubescence, shared with *E.chocoandina* sp. nov.

*Enchenopachocoandina* sp. nov. differs from *Enchenopaloranthacina* (Sakakibara & Marques, 2010) by the obtuse projection obliquely directed forwards rather than a horizontally inclined horn and from *E.pilosa* Strümpel & Strümpel, 2014 and *E.eurycephala* Strümpel & Strümpel, 2014 by the head longer than wide instead of as long as wide or wider than long respectively. *Enchenopachocoandina* sp. nov. has a reddish median pronotal carina and amber forewing patches which separate it from *E.monoceros* (Germar, 1821) and *E.luizae* (Lencioni-Neto & Sakakibara, 2015) which have the median carina concolorous and forewing patches hyaline. The new species resembles *E.andina* (Schmidt, 1924) by the black overall coloration with median carina and posterior apex reddish, and the amber forewing apex; however, *E.chocoandina* sp. nov. does not have a horn instead an obtuse projection, longer and denser pubescence, and three or four weak and irregular accessory carinae instead of two or three. Moreover, the new species is considerably shorter than *E.andina* and differs in the male and female genitalia.

*Enchenopachocoandina* sp. nov. lacks a distinctive horn; instead, it has an obtuse projection with a rounded apex, resembling species of the *E.beebi* species group or the males of *E.minuta* species group (Strümpel and Strümpel 2014). However, *E.chocoandina* sp. nov. properly does not fit within the *E.beebi* species group due to the absence of large punctation on the upper portion of the pronotum and dorsum, a short translucent apical patch and yellow tarsi. Neither does it belong to the *E.minuta* species group due to the absence of sexual dimorphism in pronotal horn shape; in *E.chocoandina* both females and males lack a horn.

We suggest *E.andina* and *E.chocoandina* sp. nov. could be related species by the scarlet median carina and posterior apex only shared in both species. Both inhabit mountain forests of the north Andes of Ecuador however are geographically separated by the Interandean Valley.

## ﻿Discussion

This study increases the number of valid *Enchenopa* species worldwide to 58 and 17 species for Ecuador. Furthermore, ten species are currently recognized within the *E.biplaga* species group and seven in the *E.andina* species group. Unfortunately, Strümpel and Strümpel (2014) did not provide specific localities from the species recorded in Ecuador; thus, based on Goding (1928), *Enchenopagennyae* sp. nov. and *E.chocoandina* sp. nov. could be the only species known from northwestern Ecuador because most species are recorded mainly in the Amazon region and a few in the Interandean Valley and central to southwestern Ecuador. Nevertheless, further studies are needed to understand better the distribution of *Enchenopa* species within Ecuador and, likely, there are still new species to be discovered.

Strümpel and Strümpel (2014) argued that *Enchenopa* is not supported by any synapomorphies but, instead a combination of characters, some of which are shared with other Membracini genera, and therefore suggested the genus is likely a paraphyletic group. Previously, Lin et al. (2004) found in their molecular phylogeny of Membracinae that *Enchenopa* is polyphyletic and even *Campylenchia* (currently synonymized in *Enchenopa*) belongs to a different clade together with the genera *Kronides* Kirkaldy, 1904 and *Tylopelta* Fowler, 1894. Likewise, species with intermediate characters have been found that cannot be assigned with confidence to any genera until the phylogenetic relationships within the tribe are resolved (Dietrich and McKamey 1995; McKamey 2022).

Because *Enchenopa* is a phenetic and probably non-monophyletic group, we suggest that the, *E.biplaga* and *E.andina* species groups, and likely the rest of the *Enchenopa* species groups could belong to independent lineages as they exhibit many important morphological differences and future phylogenetic studies might split them into different genera. The pronotum of the *E.biplaga* species group species is somewhat foliaceous with a dorsal spot and lateral band, and some species (e.g., *E.lanceolata*, *E.longimaculata*) even have short lateral carinae, resembling *Enchophyllum*. In contrast, in the *E.andina* group, the pronotum is not very compressed and mostly unicolorous with the horn short or reduced to a blunt projection, similar to some species of *Leioscyta*. The second valvulae of females also are strikingly different between both groups; species of the *E.biplaga* species group having the second valvule broad with a ventral-apical tooth, also shared with some species of *Enchophyllum* (Strümpel and Strümpel 2006), while in the *E.andina* species group, it is blade-shaped with dorsal blunt teeth shared with other *Enchenopa* species groups. It is likely the horn-shaped anterior process and the metopidial carination that defines *Enchenopa* could be homoplastic characters since they vary intra and interspecifically within *Enchenopa* and other Membracini (Richter 1954; Strümpel and Strümpel 2014). Until the phylogenetic relationships are resolved, however, the new species described in this study belong to the current definition of *Enchenopa*.

*Enchenopagennyae* sp. nov. inhabits the forest remnants of Santo Domingo, a populous city with extensive areas dedicated to monocultures and livestock around the urban area. This city, within its urban area, harbors small patches of secondary forest, mainly around water bodies, which hold a great diversity of native and endemic insect species. Several studies have shown urban green areas, such as urban forest fragments, to be valuable reservoirs of native arthropod biodiversity and these must be integrated with plans for conservation management (Watts and Larivière 2004; Philpott et al. 2014). Membracids are particularly abundant in this kind of ecosystem because they prefer sun-exposed vegetation (Wood 1993). Thus, we reiterate the importance of urban green areas for the conservation of local biodiversity and even unnamed species.

## Supplementary Material

XML Treatment for Enchenopa gennyae

XML Treatment for Enchenopa chocoandina

## ﻿Additional information

### Author ORCIDs

María P. Rueda-Rodríguez https://orcid.org/0009-0002-8190-7239

Jorge L. Montalvo-Salazar https://orcid.org/0009-0003-1221-0304

### Data availability

All of the data that support the findings of this study are available in the main text.
